# Adolescent Loneliness and Social Skills: Agreement and Discrepancies Between Self-, Meta-, and Peer-Evaluations

**DOI:** 10.1007/s10964-016-0461-y

**Published:** 2016-04-12

**Authors:** G. M. A. Lodder, L. Goossens, R. H. J. Scholte, R. C. M. E. Engels, M. Verhagen

**Affiliations:** 1 Behavioural Science Institute, Radboud University Nijmegen, Nijmegen, The Netherlands; 2 Research Group School Psychology and Child and Adolescent Development, KU Leuven, Louvain, Belgium; 3 Praktikon, Nijmegen, The Netherlands; 4 Netherlands Institute of Mental Health and Addiction, Trimbos Institute, Utrecht, The Netherlands; 5 Social and Behavioural Sciences, Utrecht University, Utrecht, The Netherlands; 6 Interuniversity Centre for Social Science Theory and Methodology, Department of Sociology, University of Groningen, Groningen, The Netherlands

**Keywords:** Loneliness, Social skills, Bias, Discrepancies, Peer evaluations

## Abstract

Lonely adolescents report that they have poor social skills, but it is unknown whether this is due to an accurate perception of a social skills deficit, or a biased negative perception. This is an important distinction, as actual social skills deficits require different treatments than biased negative perceptions. In this study, we compared self-reported social skills evaluations with peer-reported social skills and meta-evaluations of social skills (i.e., adolescents’ perceptions of how they believe their classmates evaluate them). Based on the social skills view, we expected negative relations between loneliness and these three forms of social skills evaluations. Based on the bias view, we expected lonely adolescents to have more negative self- and meta-evaluations compared to peer-evaluations of social skills. Participants were 1342 adolescents (48.64 % male, *M*_age_ = 13.95, *SD* = .54). All classmates rated each other in a round-robin design to obtain peer-evaluations. Self- and meta-evaluations were obtained using self-reports. Data were analyzed using polynomial regression analyses and response surface modeling. The results indicated that, when self-, peer- and meta-evaluations were similar, a greater sense of loneliness was related to poorer social skills. Loneliness was also related to larger discrepancies between self- and peer-evaluations of loneliness, but not related to the direction of these discrepancies. Thus, for some lonely adolescents, loneliness may be related to an actual social skills deficit, whereas for others a biased negative perception of one’s own social skills or a mismatch with the environment may be related to their loneliness. This implies that different mechanisms may underlie loneliness, which has implications for interventions.

**Electronic supplementary material:**

The online version of this article (doi:10.1007/s10964-016-0461-y) contains supplementary material, which is available to authorized users.

## Introduction

Loneliness is defined as a subjective experience of lack of connectedness, in terms of quantity or quality of social relations (Heinrich and Gullone 2006). It can have severe negative consequences for both mental and physical health, including depression, suicidal ideation, aggression, obesity, and cardiovascular diseases (Cacioppo et al. 2015), and even increases the risk for early mortality (Holt-Lunstad et al. 2015). Previous research showed that loneliness and self-evaluations of social skills are negatively related (e.g., Segrin and Flora 2000), but little is known about whether social skills evaluations from others may also be related to loneliness. For instance, it is still unknown whether lonely adolescents evaluate their social skills more positively or negatively than their peers do, and whether *perceptions* of others’ evaluations (i.e., meta-evaluations) may have a larger impact on loneliness than others’ *actual* evaluations. In addition, the studies that have examined the relationship between loneliness and social skills as reported by others have been conducted among children and adults, whereas few studies focused on adolescence (Qualter et al. 2015). Research in this age group is needed because adolescence is a crucial period for both the development of social skills and loneliness.

During early adolescence, adolescents enter the complex world of social relations that is typical for this developmental period. Peers become increasingly important during early adolescence, as adolescents become part of a complex network of friendships (Brown and Klute 2006). Moreover, peers play an important role in the development of social and emotional skills during adolescence (Steinberg and Morris 2001). As such, early adolescents may be especially sensitive to develop loneliness compared to other age groups, and the link between social skills and loneliness seems particularly worthwhile to examine in this developmental period. Indeed, prevalence of loneliness in adolescence is high, with between 21 and 70 % of adolescents feeling lonely at least sometimes (Qualter et al. 2015) and between 3 and 22 % of adolescents chronically experiencing loneliness (van Dulmen and Goossens 2013). The goal of the present study was therefore to examine whether during early adolescence, loneliness was related to adolescents’ self-, peer-, and meta-evaluations of social skills, and whether discrepancies between these types of evaluations were related to loneliness.

Social skills can be defined as the ability to operate successfully in one’s social environment (Cillessen and Bellmore 2011). Some researchers argue that loneliness is caused by a social skills deficit (Segrin and Flora 2000). According to this theory, people with low social skills have difficulties interacting with others, which limits their opportunity to form and maintain satisfactory friendships with their peers, and thereby limiting the quantity of their social relations. Moreover, if people have low social skills, they may not be able to adequately cope with stressful life events by engaging their social network, leading to increased negative affect (Segrin 1999). As such, quality of friendships may also be lower in people who have a social skills deficit. As both quantity and quality of social relations are related to loneliness in adolescence (Lodder et al. 2015), a social skills deficit may thus cause feelings of loneliness. In addition, once loneliness is experienced, further problems with social skills may develop. Loneliness can cause withdrawal from social relations, which then limits opportunities for adolescents to further develop social skills (Qualter et al. 2015). As such, problems with social skills may cause loneliness, and loneliness may be a maintaining factor for social skills problems.

Empirical studies have demonstrated that, across development, loneliness is related to lower self-reported social skills in different age groups (Qualter et al. 2015), including adulthood (DiTommaso et al. 2003) and mid-adolescence (Inderbitzen-Pisaruk et al. 1992). Concerning ratings by others, research on adults shows that findings on the relationship between loneliness and conversational skills are mixed. Jones et al. (1981, 1982, 1983) conducted several well-known studies on the relationship between loneliness and social skills, in which lonely adults were paired with others for a conversation. This research showed that, for ratings of conversation skills, lonely adults rated themselves negatively, expected negative ratings form their interaction partners, rated their interaction partners slightly negatively, but were not rated negatively by their interaction partners (Jones et al. 1981). Additional research showed that lonely males, compared to females, were also rated negatively by their interaction partners (Jones et al. 1983). Still, other research showed that loneliness was related to lower attention to interaction partners, and when lonely subjects were trained to pay more attention to their partner, their loneliness decreased (Jones et al. 1982).

In children and adolescents, research on ratings of social skills by significant others is scarce, but indicates that increasing loneliness may be related to lower mother-reported social skills (Schinka et al. 2013). In contrast, independent observers indicate that lonely as well as nonlonely children exhibit prosocial behaviors like initiating conversations, to which their peers respond well (Qualter and Munn 2005). There is evidence to suggest that loneliness is related to withdrawn and shy behavior, which some researchers argue is a sign of poor social skills. Peer reports show that loneliness was related to social withdrawal in late childhood, (Boivin et al. 1995), and to shyness in late adolescence (Woodhouse et al. 2012), and peer rated social withdrawal predicts increases in loneliness from middle to late childhood over time (Jobe-Shields et al. 2011). Finally, teacher and mother rated shyness was also related to loneliness in middle childhood (Coplan and Weeks 2010). Overall, unlike the consistent findings for the relationship between loneliness and self-reported social skills, mixed evidence exists for a negative relationship between loneliness and other-reported social skills. This indicates that loneliness may be related to an objective social skills problem to some extent, but lonely individuals may subjectively experience a much larger social skills deficit.

Indeed, some researchers have argued that individuals’ own perceptions of social functioning (Vanhalst et al. 2013), and anxiety about interactions (Solano and Koester 1989) may be more strongly reflected in feelings of loneliness than their actual social skills, and may cause lonely individuals to “choke under the pressure” of social interactions (Knowles et al. 2015). This could explain why interventions aimed to reduce loneliness by social skills training are usually not very effective, but interventions that address maladaptive cognitions are effective in reducing loneliness (Cacioppo et al. 2015).

In line with the idea that maladaptive cognitions may be related to loneliness, some research suggests that loneliness is related to hypervigilance for rejection, causing lonely individuals to focus on negative information in the social environment (Cacioppo and Hawkley 2009), which may lead to a biased negative view of the social environment (Qualter et al. 2013). In line with this idea, earlier research showed loneliness was related to having a hostile attribution bias (Qualter et al. 2013), and to a self-defeating attribution style in which social success is attributed to external factors and social failure to internal factors (Crick and Ladd 1993). In addition, chronic loneliness is related to the tendency to attribute social exclusion to internal and stable factors, and social inclusion to unstable and external factors (Vanhalst et al. 2015). Moreover, there is evidence to suggest that lonely adolescents may view the quality of their friendships more negatively than their friends do (Lodder et al. 2015), and that they show greater negative affect tin response to negative company (van Roekel et al. 2014). Overall, this pattern of findings indicates that lonely adolescents tend to negatively interpret the social environment, their relations, and their role in social relations. Possibly, this negative view does not only entail external social stimuli such as emotional expressions, but also a negative view of oneself, resulting in a negative bias towards one’s own social skills.

To examine whether loneliness is related to lower social skills, distorted negative perceptions of social skills, or both, it is necessary to compare views on social skills of adolescents themselves with others’ views on adolescents’ social skills. Researchers have argued that peer-observers may be most valuable when considering peer-related social skills, as these peers may respond to perceptions of low skills by rejecting the adolescent (Miers et al. 2011). Indeed, earlier research showed that among socially anxious adolescents, peer-reports of social skills were more closely related to adolescents self-reports of social skills compared to social skills as reported by adult observers. We, therefore, decided to use peer-observers as an indication of others-evaluation. Lonely adolescents may have a negative view of their social skills due to an actual social skills deficit. If this were the case, we would expect that loneliness should be negatively related to others’ evaluations of adolescents’ social skills as well as self-reported social skills. Alternatively, according to the bias view, loneliness may be unrelated to others’ evaluations. Rather, loneliness may be related to a discrepancy between self- and meta-evaluations of social skills on the one hand, and peer-evaluations of social skills on the other hand. Earlier research did indicate that self-, peer-, and meta-evaluations of social skills might be related to loneliness, but discrepancies between these types of evaluations have never been examined (e.g., Jones et al. 1981). The comparison of self- and meta-evaluations with peer-evaluations of social skills allows us to examine loneliness in relation to *over*-*estimation*, which occurs when lonely individuals think that others evaluate them more positively and rate themselves more positively than others actually do, and *under*-*estimation*, which occurs when lonely individuals think others evaluate them more negatively and rate themselves more negatively than others do. This comparison is relevant, because based on the bias view of loneliness, we would expect that lonely adolescents underestimate how others evaluate their social skills (Qualter et al. 2013).

A biased perception of social skills becomes apparent in the *direction* of the discrepancy between self- or meta-evaluations and peer-evaluation of social skills. Recent studies have suggested that the *size* of the discrepancies between informants’ evaluations of behavior may have a unique effect on various outcomes, beyond the main effects of the individual informants’ evaluations (De Los Reyes 2011), for instance, on aggression (Brendgen et al. 2004) and depression (Ehrlich et al. 2014). As of yet, no studies have examined the possible relationship between loneliness and informant discrepancies, which is important because the bias hypothesis implies discrepancies between perceived and actual social functioning (Qualter et al. 2013).

## Current Study

In the current study, we examined whether loneliness was related to social skills as evaluated by adolescents themselves and by their peers, and to perceptions adolescents had about how their peers would evaluate them (meta-evaluations). Moreover, we examined whether the size and direction of discrepancies between self-, peer- and meta-evaluations of social skills would be related to loneliness. Different hypotheses for these relations can be formulated based on the social skills deficit view (e.g., Segrin and Flora 2000) and the bias view (e.g., Qualter et al. 2013). Based on the social skills deficit view, we hypothesized that lonely adolescents would show limited social skills. This would result in a negative relationship between loneliness on the one hand, and self-, peer-, and meta-evaluations of social skills on the other hand. Based on the social skills deficit view, we would not expect a discrepancy between self-, peer- and meta-evaluations of social skills. That is, if there actually is a social skills deficit, one would expect that self-, peer- and meta-evaluations of social skills would be negative, and would not differ from each other. Based on the bias view, we expected that loneliness would be related to an underestimation of social skills. This would be evidenced by a discrepancy between self- and meta-evaluations of social skills on the one hand, and peer-evaluations of social skills on the other hand. Based on the bias view, we would expect that this discrepancy would contribute to loneliness over and above the main effects of the individual informants.

## Method

### Participants and Procedure

Six secondary schools in The Netherlands agreed to participate after receiving information about the study through written and personal communication. The data were collected from 1342 participants (48.64 % male), in February–April 2014. For each participant, passive parental consent and active consent from adolescents was obtained. The IRB of the faculty of social sciences approved the study procedures (ECG2012-2711-701). Of the 1467 students that were enrolled in the schools at the time the data were collected, 80 (5.45 %) adolescents were not present, 39 (2.66 %) did not have parental consent to participate, and 6 adolescents (0.41 %) did not assent to participate themselves. Due to time constraints (e.g., a shortened class schedule), not all participants were able to complete all questionnaires (see Table 1). Participants were all in the second grade of secondary education, and were 13.94 years old on average (*SD* = 0.47). Most participants had a Dutch ethnic background (96.4 %). In the Dutch school system, students follow different educational paths, ranging from low (i.e., pre-vocational level) to high (i.e., pre-university level). In our sample, 22.7 % of the students attended a low to middle level of education, 38 % attended a middle to high level of education, and 39.3 % attended a high level of education. Participants completed all measures during regular school hours on a computer under the supervision of undergraduate students involved in the project.

### Measures

#### Loneliness

Loneliness was measured using the peer-related subscale of the Louvain Loneliness and Aloneness Scale for Children and Adolescents (LACA; Marcoen et al. 1987). The 12 items on this scale can be answered on a 4-point scale ranging from *never* (1) to *always* (4) (e.g., “I feel alone at school”). Cronbach’s alpha was .91.

#### Evaluations of Social Skills

Self-, peer-, and meta-evaluations of social skills were each measured using three items that referred to *being nice and helping others*, *being good at making friends,* and *being cooperative*. These items were designed to measure the components of social skills as defined by Cillessen and Bellmore (2011) as being prosocial and cooperative, and being interpersonally successful. All evaluations were measured on a 6-point scale ranging from 1 (*not at all*) to 6 (v*ery much*). For self-evaluations, participants reported on their own social skills (e.g., “Are you good at making friends?”) (α = .79). For meta-evaluations, participants indicated how they thought their classmates would evaluate them (e.g., “Do you think your classmates think you are somebody who is good at making friends?”) (α = .85).

In the Dutch school system, adolescents have a designated group with whom they take most of their classes. Class size varied from 17 to 31 (*M* = 23.96, *SD* = 3.37). Peer-evaluations were based on the average rating participants received from their classmates (e.g., “Do you think Sam is somebody who is good at making friends?”). Although round robin data can typically be examined using the Social Relations Model (SRM; Back and Kenny 2010), this technique was not suitable for our research purposes. With the SRM, variance due to peer evaluations (i.e., partner effects) can be partialled out, controlled for the tendencies of individual raters to see others in a certain way (i.e., actor effects), and the relationship between specific individuals (i.e., relationship effects) in a classroom. However, to compare the fit between individuals and their environment (e.g., self- and peer-evaluated socials sills), it is necessary to use commensurate measures in terms of nominal and scale equivalence (Edwards 2007). Because the scale of partner-evaluations derived from SRM would be very different from the scale for self- and meta-evaluations, we decided not to use SRM to analyze peer-evaluations. Rather, we averaged the scores adolescents received from their classmates for each question and computed an average social skills score (α = .93). The correlation between partner-effects and average peer-evaluation scores was very high (*r* = .87, *p* = < .001).

### Strategy of Analyses

#### General Statistical Approach to Discrepancy Analyses

A common method to examine whether discrepancies between different reports are related to a certain outcome is the use of difference scores (e.g., to subtract the standardized score for self-evaluations from the standardized score for peer-evaluations, and regress the resulting scores on loneliness). However, the use of difference scores in a regression to assess discrepancies among observers gives rise to a number of statistical problems (Edwards 2002). For instance, given the difference score (X − Y) in relation to an outcome variable Z, the reliability of this score is usually much lower compared to the reliabilities of X and Y. Moreover, using this difference score places mathematical constraints on the relation between the two elements of a difference score and the outcome. For instance, concerning the effect of (X − Y) on Z, the effects of X and Y on Z are constrained to be equal in size but in the opposite direction. However, many researchers do not intend to impose this constraint. In our case, for instance, we wanted to examine if the difference between self- and peer-evaluations of social skills is related to loneliness, but we did not assume that the effects of self- and peer-evaluations were equal in size but in opposite indirection. By using polynomial regression, combined with response surface modeling, one can simultaneously estimate the effects of *agreement* between X and Y (i.e., what happens to Z if X and Y are similar) and the *size* and *direction* of disagreement between X and Y on an outcome Z. The advantages of this methodology have been described in greater detail in earlier research (Laird and De Los Reyes 2013).

#### Steps in the Discrepancy Analyses

We adopted the procedures used in earlier work for the discrepancy analyses (Edwards 2002; Shanock et al. 2010). Prior to the analyses, we centered self-, meta- and peer-evaluations around the scale midpoint (i.e., 3.5). In order to ensure that influential cases or multivariate outliers do not affect the results, we followed Edwards’ (2002) suggestion to remove cases that exceed the cut-off points for leverage (leverage > 2n + 2), Cook’s distance (Cook > 4/n) and standardized residual outliers (residuals > 2). In the regression of the main effects (self, peer, and meta) on loneliness, 15 cases (1.12 %) were dropped and 9 cases (0.67 %) were dropped in the discrepancy analyses.

Before we analyzed discrepancies between self-, peer- and meta-evaluations of social skills in relation to loneliness, we first examined whether each of the evaluation types was uniquely related to loneliness. To test this, we used a regression model with the main effects of each evaluation type as predictor. Second, we tested whether agreement and discrepancies between each evaluation type actually occurred in our sample. In accordance with earlier research, we considered two types of evaluation to be in agreement when their standardized measures were within half a standard deviation of each other, otherwise we considered them to be in disagreement (Shanock et al. 2010). For instance, for self-peer discrepancies, we examined the percentage of the cases with self-evaluation scores more than half a standard deviation above or below the peer-evaluation scores and the percentage of the cases with similar scores.

After checking whether each of the evaluation types uniquely related to loneliness, and testing whether agreement and discrepancies between each evaluation type actually occurred in our data, we estimated a polynomial regression model. That is, we tested a regression model with three main-effects (i.e., self-evaluations, meta-evaluations, and peer-evaluations), the square values for each predictor (i.e., self^2^, meta^2^, and peer^2^), and the interactions between these predictors (i.e., self by peer, self by meta, and meta by peer interactions) regressed on loneliness. In accordance with earlier literature on the effects of discrepancy scores, we did not interpret the outcome of this regression analysis directly but evaluated the fit of this model and used the output to examine the shape of the response surface corresponding to this model (Shanock et al. 2010).

**Fig. 1:**
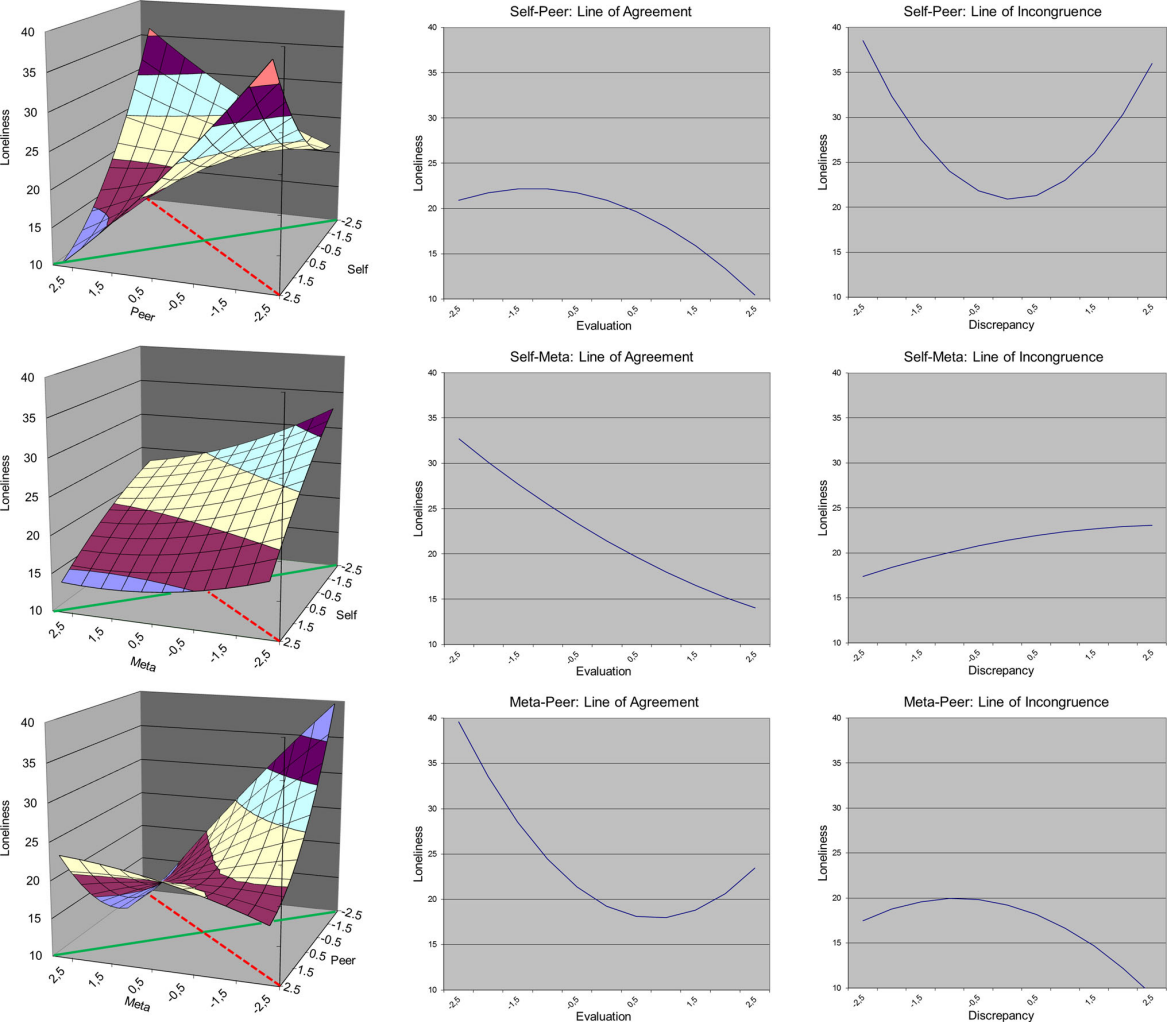
Response surfaces and graphs for the line of perfect agreement and the line of incongruence. The *top figure* displays self-peer discrepancies, the *middle figure* displays self-meta discrepancies, and the *bottom figure* displays meta-peer discrepancies. The *solid lines* at the floor of the graphs represent the line of perfect agreement, the *dashed lines* at the floor of the graph represent the line of incongruence

We plotted three response surfaces (i.e., self- vs. peer-evaluations, self- vs. meta-evaluations and meta- vs. peer-evaluations). As an example, we describe the points of interest in this plot for the self- and peer-evaluation discrepancy. First, we examined slope and curvature along the *line of perfect agreement* (the solid lines at the floor of the graphs in Fig. 1). This line describes loneliness for adolescents whose self- and peer-evaluations are similar (e.g., a low score on both self- and peer-evaluations of social skills). Second, we examined the *line of incongruence* (the dashed lines at the floor of the graphs in Fig. 1). This is the line in the plot along which the difference between self- and peer-evaluations increases. The slope (*a*1) along the line of perfect agreement indicates the effect of agreement between self- and peer-evaluations of social skills on loneliness. The curvature (a2) along the line of perfect agreement indicates whether this relation is stronger for certain values of social skills (e.g., the relation with loneliness is stronger for low evaluations of social skills compared to high evaluations of social skills). The slope along the line of incongruence (*a*3) indicates the effect of the *direction* of the difference between self- and peer-evaluations on loneliness (e.g., loneliness increases when self-evaluations are lower than peer evaluations). The curvature along the line of incongruence (*a*4) indicates the *degree* to which the difference between self- and peer-evaluations on loneliness is related to loneliness (e.g., loneliness increases as the difference between self- and peer evaluations increases).

## Results

### Descriptives

In Table 1, means and standard deviations, and correlations among all constructs are presented. All types of evaluations were related to loneliness, and to each other. A regression analysis showed that self-evaluations (b = −1.98, SE b = .29, *p* ≤ .001), meta-evaluations (b = −1.13, SE b = .27, *p* ≤ .001), and peer-evaluations (b = −.92, SE b = .29, *p* = .001) were each negatively related to loneliness (F [3,1314] = 102.44, *p* ≤ .001; adjusted *r* ^2^ = .19). Tolerance and variance inflation factor (i.e., VIF) were within acceptable range for all predictors (tolerance > .40 and VIF < 2.46 for all predictors), indicating that no problems with multicollinearity occurred.

**Table 1:** Sample size, means, standard deviations and correlations for loneliness and social competence evaluation

Measure	Descriptives			Correlations		
	*N*	*M*	*SD*	Self	Meta	Peer
Loneliness	1340	18.08	6.18	−.46***	−.45***	−.25***
*Social competence*						
Self-evaluation	1338	4.79	0.78		.76***	.29***
Meta-evaluation	1338	4.35	0.87			.34***
Peer-evaluation	1335	4.27	0.54			

*Self* Self-evaluation, *Meta* meta-evaluation, *Peer* peer-evaluation

*** *p* < .001

Next, we tested whether disagreement and agreement between self-, peer- and meta-evaluations of social skills actually occurred within our sample. The results indicated that all types of discrepancies were effectively found. For instance, some adolescents had *higher* self- than peer-evaluations, some adolescents had *lower* self- than peer-evaluations, and some adolescents had *similar* self- and peer-evaluations of social skills. For a detailed overview of the occurrence of agreement and disagreement within the sample, see Supplementary Table 1. Because all types of evaluation were uniquely related to loneliness, and because agreement and discrepancies between all types of evaluation occurred within our sample, we continued with the main analyses to examine the effect of agreement and disagreement between evaluations on loneliness.

### Discrepancy Analysis

We examined the relationship between loneliness and the discrepancies between self- meta-, and peer-evaluations of social skills (see Supplementary Table 2). Because model fit was good (*F* [9,1311] = 41.99, *p* ≤ .001; adjusted *r* ^2^ = .22, *p* ≤ .001), we proceeded to use the output of the regression model to estimate surface plots. The results of this analysis are displayed in Table 2.

**Table 2:** Shape of the response surface for all discrepancy pairs

Parameter	Self-peer discrepancy		Self-meta discrepancy		Meta-peer discrepancy	
	T	SE (B)	B	SE (B)	B	SE (B)
*Line of perfect agreement*						
Slope (a1)	−2.09*	1.01	−3.73***	0.40	−3.23***	0.67
Curve (a2)	−0.84	0.67	0.31	0.17	1.97***	0.48
*Line of incongruence*						
Slope (a3)	−0.50	0.76	1.14	1.18	−1.64	0.98
Curve (a4)	2.62***	0.74	−0.19	0.76	−0.94	0.80

* *p* ≤ .05; ** *p* ≤ .01; *** *p* ≤ .001

We estimated surface values for the line of perfect agreement and the line of incongruence separately for self-peer discrepancies, self-meta discrepancies, and meta-peer discrepancies. The response surfaces are shown in Fig. 1. The results indicated that for all analyses, the slope of the line of perfect agreement (*a*1) was negative and significant. Thus, for all discrepancy pairs, loneliness was high when both sources of evaluation agreed that social skills were low. For meta-peer discrepancies, we also found a significant positive curve for perfect agreement (*a*2). Figure 1 shows that the relationship between loneliness and evaluations of social skills was stronger for negative evaluations than for positive evaluations. Next, we examined the line of incongruence. The only significant effect that emerged was a significant curve for the discrepancy between self- and peer-evaluations (*a*4). Thus, loneliness was high when self-evaluations are either higher or lower than peer-evaluations of social skills. No significant slopes (*a*3) or curves (*a*4) were found for self-meta discrepancies or meta-peer discrepancies. The latter findings indicated that the size and direction of discrepancies between self- and meta-evaluations and the size and direction of discrepancies between meta- and peer-evaluations were not related to loneliness.

## Discussion

Loneliness is a prominent problem in early adolescence (van Dulmen and Goossens 2013). Earlier research indicated that loneliness is related to self-reported social skills (e.g., DiTommaso et al. 2003). Yet, it is unclear why loneliness may be related to self-reported social skills. According to the social skills deficit view (Segrin and Flora 2000), lonely adolescents may report lower social skills because they actually have lower social skills. Low social skills may limit opportunities to form and maintain friendships, both in terms of quality and in terms of quantity, thereby leading to social isolation and in turn to loneliness. In contrast, the bias view on loneliness states that lonely adolescents negatively interpret their social environment (Qualter et al. 2013). According to this view, lonely adolescents may report that they have low social skills, because they negatively interpret their own functioning in their social environment. Because most studies have not reported loneliness in relation to social skills as reported by others, it is difficult to determine whether lonely adolescents’ views reflect the views of their environment or not. The goal of the present study was to examine whether loneliness in adolescence is related to social skills as reported by adolescents themselves and their peers, and to ideas adolescents have about how their peers evaluate them (meta-evaluations). In addition, we examined whether discrepancies between self, peer-, and meta-evaluations of social skills were related to loneliness.

Our results indicated that loneliness was uniquely related to both self-, peer- and meta-evaluations of social skills. For each evaluation pair, we found that, if evaluations were in agreement, reports of poorer social skills were related to stronger feelings of loneliness. In addition, higher levels of loneliness were reported when a discrepancy between self- and peer-evaluations of social skills was present, but it did not matter whether self-evaluations were more negative than peer-evaluations or vice versa. Our findings are in line with both the notion that loneliness relates to poorer social skills (Segrin and Flora 2000), and the notion that loneliness relates to a biased perception of social skills (Qualter et al. 2013). Yet, our results should be interpreted with care as we cannot draw conclusions about causality in the relationship between loneliness and social skills.

In line with the social skills deficit view, our findings indicate that loneliness is negatively related to peer-evaluations of social skills after taking into account the multivariate effects of self-evaluations and meta-evaluations of social skills. Thus, contrary to earlier findings regarding social status (Vanhalst et al. 2013), our results indicated that it is not just adolescents’ perception of their own social skills (i.e., self-evaluations) or perceptions of how others evaluate them (i.e., meta-evaluations) that are related to loneliness. Rather, some lonely adolescents are evaluated negatively by their peers. In line with this, our findings indicated that, if self- and peer-evaluations of social skills were in agreement, negative evaluations were related to a greater sense of loneliness. Thus, when adolescents have a realistic and negative view of their social skills, they may be lonelier, or vice versa.

In line with the bias hypothesis, we found that discrepancies between self-and peer-evaluations of social skills were related to loneliness. Thus, if adolescents thought that they had better or poorer social skills compared to how they were evaluated by their peers, they were lonelier. The finding that adolescents may be lonelier if they evaluate themselves more negatively than their peers could reflect the fact that some lonely adolescents have a biased negative perception of their own social skills, in line with what was suggested in earlier research (Qualter et al. 2013). However, we found no evidence for a discrepancy between meta- and peer-evaluations, which would have indicated an overall biased negative perception. Alternatively, the self-peer discrepancy could reflect the fact that peers evaluate adolescents in the school context, whereas the adolescents may consider their skills in a broader context. Especially if lonely adolescents indeed tend to withdraw from social interactions (Qualter et al. 2015), their peers may not have a nuanced view of lonely adolescents’ skills. Future research could therefore expand the present research by including reports on social skills by other informants, such as friends or mothers.

Unexpectedly, we found that when they reported that their social skills were better than what was reported by their peers, adolescents were also lonelier. Possibly, some lonely adolescents think that they have appropriate social skills because they know how to act in social situations, but they are unable to apply this knowledge in actual social situations. This idea is in line with the social monitor theory (Gardner et al. 2005), and recent research suggested that lonely people may have appropriate social skills in terms of knowing how to act in social situations, but choke under the pressure of actual social situations (Knowles et al. 2015). Additionally, peers might reject classmates whom they believe have poor social skills, resulting in social isolation of the adolescent and consequently in feelings of loneliness, even if adolescents themselves believe that this negative evaluation is unfounded. Alternatively, the discrepancy between self- and peer-evaluations of social skills may represent a mismatch between adolescents and their environments. Earlier research suggested that informant discrepancies may have an effect on problem behavior beyond the effects of the individual informant (De Los Reyes 2011). This mismatch may cause loneliness, or loneliness may cause a mismatch with the environment. Future research could explore this possibility by examining loneliness in relation to self-peer discrepancies for other constructs such as social interests and general world view. Moreover, future research could incorporate objective measures of social skills in multiple settings, to examine whether the views of adolescents and their peers reflect actual social skills.

Our study was the first to not only look at the unique effects of self-, peer- and meta-evaluations of social skills on loneliness, but also at the discrepancies between each of these types of evaluations. This allowed us to examine whether the negative relationship between loneliness and self-reported social skills that was found in earlier research (e.g., DiTommaso et al. 2003) was also reflected in social skills evaluations of peers, or whether only lonely adolescents themselves report poor social skills. Another strength of the present study was that we used a powerful method, which allowed us to overcome the shortcomings of difference scores and provided greater insight into the interplay between self- peer-, and meta-evaluations of social skills in relationship to loneliness (Edwards 2002). In addition, we used a round-robin design to measure peer-evaluations, which resulted in a more detailed measure of peer-evaluations compared to nomination procedures.

This study also had a few limitations. One limitation of the present study is that we used a general measure of meta-evaluations of social skills, rather than a round robin design with meta-evaluations for each individual classmate. Future research could incorporate such an individualized design, which would also allow scholars to examine meta- and peer-evaluations for specific types of classmates such as friends, bullies, or popular peers. Another limitation is that our study was correlational, which makes it impossible to determine whether loneliness is a cause or consequence of poor social skills, and discrepancies between self- and peer-evaluations of social skills. A third limitation is that we focused mainly on pro-social skills, and utilized a global behavioral trait approach to social skills (cf., Dirks et al. 2007). Future research could benefit from utilizing other measures of social skills, such as situation-based measurements (Dirks et al. 2007). The use of such a measure also decreases the likelihood that adolescents rate their own social skills in a broader context, whereas they rate the skills of their peers only related to the school context. In addition, besides prosocial behavior, social skills include a wide range of traits and behaviors (McFall 1982). Future research could examine discrepancies between informant reports on other social skills that have been related to loneliness, including withdrawn behavior (Qualter et al. 2015) and negative behavioral tendencies such as aggression, narcissism, and Machiavellianism (Zhang et al. 2015). A final limitation of the present study is that we used self-reported measures for loneliness, self-evaluations, and meta-evaluations, which causes shared method variance. We believe that self-reports are necessary, because loneliness, self-evaluations, and meta-evaluations are each subjective in nature. Nevertheless, future research could explore the effects of self-, meta-, and peer-evaluations of social skills on other measures, such as peer-reported loneliness (i.e., social isolation).

## Conclusion

Our findings indicate that there may be different mechanisms underlying loneliness. First, for some adolescents, as suggested by the social skills deficit view (Segrin and Flora 2000), loneliness may be related to having poor social skills. Our findings indicated that poor social skills, as evaluated by adolescents themselves, their peers, and ideas adolescents have about how their peers evaluate them, are each related to a greater sense of loneliness. In addition, our discrepancy analyses showed that, when self- and peer-evaluations were in line, poorer social skills evaluations were related to more pronounced feelings of loneliness. Second, for other adolescents, as suggested by the bias view (Qualter et al. 2013), loneliness may be related to a negative bias of one’s own social functioning, evidenced by our finding that if they rated their social skills more negatively compared to their peers, adolescents felt lonelier. Our findings also indicated that when adolescents rated their social skills more positively than their peers did, they also felt lonelier. This indicates that, third, loneliness may be related to a mismatch between adolescents and their environment. Future research could explore the idea that different social skills are related to loneliness through different mechanisms using a longitudinal design with a person-centered approach. Future research could also explore whether the same mechanisms are observable in different age groups. If indeed different mechanisms underlie loneliness, this has implications for interventions. Some adolescents might benefit from social skills training, whereas others might benefit from a cognitive approach, which has proven to be successful in reducing loneliness (Cacioppo et al. 2015). Screening adolescents to examine whether they have a realistic (negative) view of their social skills, or whether their view differs from that of their peers, could help tailoring interventions to the specific needs of each adolescent.

## Electronic supplementary material

Below is the link to the electronic supplementary material.

Supplementary material 1 (DOCX 25 kb)
