# Supplementary material for: Adolescent Loneliness and Social Skills: Agreement and Discrepancies Between Self-, Meta-, and Peer-Evaluations
Source: J Youth Adolesc. 2016 Apr 12;45(12):2406–16. doi: 10.1007/s10964-016-0461-y (PMC5101254; doi:10.1007/s10964-016-0461-y)
Supplement: Supplementary file 1 — Supplementary material 1 (DOCX 25 kb) [file 10964_2016_461_MOESM1_ESM.docx]

Supplementary Table 1

*Occurrences of Agreement and Disagreement Between Self-, Peer, and Meta-Evaluations of Social Skills*

| Type | Percentage | Mean X | Mean Y |
| --- | --- | --- | --- |
| Self vs. Peer |  |  |  |
| Self > Peer | 31.6% | 5.25 | 3.87 |
| Self = Peer | 34.4% | 4.90 | 4.34 |
| Self < Peer | 34.0% | 4.24 | 4.56 |
| Self vs. Meta |  |  |  |
| Self > Meta | 20.0% | 5.07 | 3.80 |
| Self = Meta | 60.1% | 4.90 | 4.53 |
| Self < Meta | 19.9% | 4.16 | 4.37 |
| Meta vs. Peer |  |  |  |
| Meta > Peer | 31.8% | 4.86 | 3.88 |
| Meta = Peer | 34.8% | 4.44 | 4.33 |
| Meta < Peer | 33.4% | 3.78 | 4.56 |

*Note*. Mean X and Mean Y refer to the means of the first comparison group and the second comparison group, for instance, for the Self – Peer discrepancy, Mean X refers to mean of Self, and Mean Y refers to the mean of Peer.

Supplementary Table 2

*Polynomial Regression Results for the Effect of Self-, Peer, and Meta-Evaluations of Social Competence on Loneliness*

| Effect | B | SE (B) |
| --- | --- | --- |
| Intercept | 21.81 | 0.44 |
| Self | -1.30 | 0.66 |
| Peer | -0.79 | 0.60 |
| Meta | -2.43*** | 0.59 |
| Self Squared | 0.22 | 0.30 |
| Peer Squared | 0.67 | 0.39 |
| Meta Squared | -0.16 | 0.21 |
| Self by Peer Interaction | -1.73** | 0.51 |
| Self by Meta Interaction | 0.25 | 0.40 |
| Meta by Peer Interaction | 1.45** | 0.48 |

*Notes.* Regression effects are not interpreted, but used as input for Response Surface (see Table 4). ** *p* = < .01. *** *p* = < .001
